# Psychological correlates of perceived loneliness in college students before and during the COVID-19 stay-at-home period: a longitudinal study

**DOI:** 10.1186/s40359-023-01099-1

**Published:** 2023-03-06

**Authors:** Chiara Conti, Roberta Lanzara, Ilenia Rosa, Markus M. Müller, Piero Porcelli

**Affiliations:** 1 Department of Psychological, Health, and Territorial Sciences, University “G. d’Annunzio” of Chieti-Pescara, Chieti, Italy; 2 Department of Dynamic and Clinical Psychology, and Health Studies, Sapienza” University of Rome, Via Degli Apuli, 1, 00185 Rome, Italy; 3 Department of Psychosomatic Medicine and Psychotherapy, Paracelsus Medical University, Nuremberg General Hospital, Nuremberg, Germany

**Keywords:** Anxiety, Depression, Somatic symptoms, Psychological distress, Alexithymia, SARS-CoV-2

## Abstract

**Background:**

Loneliness is increasingly acknowledged as a serious public health issue. This longitudinal study aimed to assess the extent to which psychological distress and alexithymia can predict loneliness among Italian college students before and one year after the COVID-19 outbreak.

**Methods:**

A convenience sample of 177 psychology college students were recruited. Loneliness (UCLA), alexithymia (TAS-20), anxiety symptoms (GAD-7), depressive symptoms (PHQ-9), and somatic symptoms (PHQ-15) were assessed before the COVID-19 outbreak and one year after the spread of COVID-19 worldwide.

**Results:**

After controlling for baseline loneliness, students with high levels of loneliness during lockdown showed worsening psychological distress and alexithymic traits over time. Suffering from depressive symptoms before COVID-19 and the aggravation of alexithymic traits independently predicted 41% of perceived loneliness during the COVID-19 outbreak.

**Conclusions:**

College students with higher levels of depression and alexithymic traits both before and one year after the lockdown were more at risk of suffering from perceived loneliness and may constitute the target sample for psychological support and intervention.

## Background

Loneliness is increasingly recognized as the next critical public health issue [1]. Loneliness, or perceived social isolation, is defined as a subjective experience where one perceives a discrepancy between their actual and desired levels of social relationships [2]. While isolation or living alone contributes to subjective loneliness, this latter is distinct from objective social isolation, which in turn is defined as the number of social connections with persons in the proximal individual environment [3]. It is estimated that more than 40% of all-age Americans [4] and 10–30% of children aged 10–15 in the UK [5] experienced a significant sense of loneliness.

Loneliness has been found to impact both physical and mental health. For example, longitudinal studies have found loneliness to predict future occurrence of depression and anxiety [6, 7] as well as medium-to-large associations of loneliness with several clinical outcomes as psychopathology, severe somatic symptoms, suicidality, general health, cardiovascular morbidity and mortality, cognitive decline, dementia, altered sleep–wake balance, increased pro-inflammatory activity, chronic comorbidity, and cardiovascular- and cancer-related mortality [1, 8, 9]. Currently, there is inadequate causal evidence of the predictive factors of loneliness but associations with predisposing personality traits like low self-esteem, shyness, introversion, self-consciousness, resilience, or optimism have been established [10, 11]. Moreover, societal forces and cultural factors have been associated with loneliness [12]. A recent worldwide study investigating loneliness in more than 46,000 persons aged 16–99 living in 237 countries found that loneliness is strongly affected by individualism-bounded (as opposed to collectivism-bounded) culture, particularly in young men [13].

Assessments of loneliness over the lifespan identified high risk for loneliness amongst the population engaged in significant life transitions, including children leaving the home, retirement, death of a spouse, and physical ailments [14], all of which may contribute to feeling lonely [2]. For example, in a large study on primary care patients, perceived loneliness was found to be strongly associated with age, with 33% of individuals aged < 25 reporting a high sense of feeling alone compared to 11% of individuals aged > 65 [15]. Adolescence and early adulthood are periods of significant life transitions, including academic and vocational decisions and leaving one’s childhood home, which may directly increase feelings of loneliness or impact feelings of depression or anxiety that in turn may increase perceived loneliness. Being under a lot of pressure to perform academically, college students are prone to developing mental health problems [16–19] and concerns about their social networks [20, 21]. Students can experience high levels of social contact but still feel lonely due to cognitive discrepancies between the desired quality of relationships and their actual experiences [22, 23]. Earlier studies have demonstrated a strong connection between college students’ experience of loneliness and depression and anxiety [23, 24].

The COVID-19 pandemic, among many other issues, has had a profound impact on students’ social life and social relationships. Furthermore, there are studies that highlight how issues such as social crises (e.g., as a result of an epidemic or migration) and the nature of our communication (e.g., digital communication and the use of social media) are related to the development and maintenance of loneliness [25, 26]. For example, changes in social networks due to social distancing caused by the COVID-19 crisis may lead to poorer mental health [27] and higher loneliness [10, 28]. COVID-19 has placed a strain on healthcare systems in significant although obvious ways (dramatic higher hospitalizations, mortality rates, reorganization of scheduled medical visits and interventions for chronic patients, increased socioeconomic costs, etc.) [e.g., 29]. Beyond these immediate aspects, COVID-19 posed a profound threat to our basic need for human connection. In the last year, peer-reviewed journals published many editorial and commentary articles expressing concern about the potential impact of the COVID-19 pandemic on isolation and resulting loneliness among the worldwide population [e.g., 10, 30–33]. By March 2020, like most countries worldwide, Italy had implemented social distancing, and the shutdown of non-essential businesses, schools, and community gathering places. As universities suspended classroom teaching and switched to online teaching, university staff worked from home, and all buildings were closed, the lives of college students changed drastically. Cross-sectional data collected early in the pandemic period suggested that college students were disproportionately affected by stress, depression, anxiety, and loneliness due to reduced social interactions, lack of social support, and newly arising stressors associated with the COVID-19 crisis [28, 34–37]. Despite distress and loneliness, personal resilience, positive coping behavior, and adequate social support were factors useful to cope with the burden associated with the pandemic [38–40]. This suggests that the students' adaptive capacity depends upon their individual and psycho-social characteristics.

Some few evidence suggested that alexithymia may be one of the psychological factors involved in the psychological health during COVID-19 pandemic [41]. Alexithymia is a multifaceted personality dimension composed of two higher‐order factors: a deficit of affect awareness (difficulty identifying feelings [DIF] and difficulty describing feelings [DDF]) and operatory thinking (externally oriented thinking [EOT] and poor imaginal processes), that are thought to reflect difficulties in cognitive processing of feelings and emotion regulation [42]. Alexithymia has been proposed as a personality trait that greatly undermines relational quality [43]. Individuals with high levels of alexithymia, probably due to impaired intimate communication, report poorer social support networks and feelings of disconnection from others [44–46]. For example, Chinese home-quarantined university students during the COVID-19 pandemic with probable depression or post-traumatic stress disorder reported more severe signs of alexithymia [47] and, in a Turkish study, adults with good social relationships showed lower levels of rumination and alexithymia than those living alone [48]. Also, in a pre-pandemic study on UK undergraduate psychology students, a direct association was found between alexithymia and loneliness, suggesting that a reduced capacity to talk about and describe one’s feelings leads to interpersonal problems that make difficult the formation of key relationships for the well-being of the individual [49].

Identifying subjects who are at risk of higher perceived loneliness and related psychological factors is particularly important in the context of the so called “long COVID” syndrome [50], so that mental health promotion efforts can target the most vulnerable individuals. We conducted a longitudinal study to assess the extent to which psychological distress and alexithymia can predict perceived loneliness among Italian college students before (T0) and one year after the COVID-19 outbreak (T1).

## Methods

### Study design

The present study is a longitudinal research with a twofold aim: (a) to investigate whether students with higher loneliness during COVID-19-related lockdown have higher distressing symptoms (i.e., depressive, anxiety, and somatic symptoms) and alexithymic traits over time as compared with students with lower loneliness; and (b) to explore whether and to what extent psychological features and their change over time are associated with higher loneliness during COVID-19-related lockdown. Based upon the previous literature, we expected that: (a) college students with higher perceived loneliness during the COVID-19 pandemic would exhibit higher levels of psychological distress and alexithymia traits over time than students with lower loneliness; and (b) psychological status before the COVID-19 pandemic and its change over the course of pandemic would predict loneliness during the COVID-19-related lockdown.

### Participants and procedure

The study was conducted at the University of Chieti, Italy, among psychology students. From a larger database of students assessed during 2019 for a research project on individual academical resources, a convenience sample of 185 subjects entered the study. The sample was formed of students whose data were collected immediately before the COVID-19 outbreak (T0, October to December 2019) and contacted one year after the spread of COVID-19 worldwide, during the second COVID-19 pandemic peak and stay-at-home period in Italy (T1, March to May 2021). Students were consecutively enrolled during university classes and were invited through email to complete an online survey (www.qualtrics.com/it) on both occasions and received course credits for their participation.

Inclusion criteria were being regular university students of Psychological Sciences or Clinical and Health Psychology courses and having a good understanding of the Italian language. Exclusion criteria were self-reported severe medical, psychiatric, or neurological disorders in the last 5 years and inability to complete the questionnaire. After removing those who did not meet the inclusion criteria or provided incomplete data, the final sample consisted of 177 (95.7%) students who completed all the questionnaires at both time points.

All participants provided online informed consent to take part in the study. The study was designed and carried out in accordance with the World Medical Association Declaration of Helsinki and its subsequent revisions [51] and approved by the Ethics Committee of the Department of Psychological, Health and Territorial Sciences (DiSPuTer) of University G. d’Annunzio—Chieti-Pescara (Protocol Number: 20003).

### Measures

*Sociodemographic and COVID-19-related characteristics*. Ad-hoc questions concerning sociodemographic variables were included in the online survey. Data were self-reported by the participants, including gender, age, and student position. COVID-19 positivity was assessed with a single item by asking participants whether they were currently or had been positive for COVID-19 in the last year. Answers were based on three choices: “yes, with severe symptoms”, “yes, with mild symptoms or asymptomatic”, and “no”.

*Loneliness.* Loneliness was measured using the Italian version of 20-item UCLA (University of California, Los Angeles) Loneliness Scale—version 3 [52, 53]. Participants were asked to rate how often loneliness-related feelings were descriptive for them (e.g., “How often do you feel that you are “in tune” with the people around you?”; “How often do you feel that your relationships with others are not meaningful?”). Each item is scored on a four-point Likert scale from 1 (“never”) to 4 (“often”). The total score can range between 20 and 80. Higher scores indicate a greater perception of loneliness. Within this sample, Cronbach’s α was 0.94 (T0) and 0.95 (T1).

*Depressive symptoms*. Depressive symptoms were measured using the Italian version of 9-item Patient Health Questionnaire (PHQ-9) [54, 55]. Participants were asked to rate how often they have been bothered by each symptom during the last two weeks. Each item is scored on a four-point Likert scale from 0 (“not at all”) to 3 (“nearly every day”). The total score can range between 0 and 27. Scores of 5, 10, 15, and 20 indicate cutoff points for mild, moderate, moderately severe, and severe depressive symptoms, respectively. Scores of > 9 could indicate clinical depression [56]. Within this sample, Cronbach’s α was 0.85 (T0) and 0.88 (T1).

*Anxiety symptoms*. Anxiety symptoms were measured using the Italian version of 7-item Generalized Anxiety Disorder scale (GAD-7)[57].^1^ Participants were asked to rate the severity of each symptom during the last two weeks. Each item is scored on a four-point Likert scale from 0 (“not at all”) to 3 (“every day”). The total score can range between 0 and 21, with higher scores indicating greater anxiety symptomology. GAD-7 scores of 5, 10, 15, represent cutoff points for mild, moderate, and severe anxiety symptoms, respectively. Scores of > 9 could indicate a generalized anxiety disorder [57]. Within this sample, Cronbach’s α was 0.88 (T0) and 0.90 (T1).

*Somatic symptoms*. The burden of somatic symptoms was measured using the Italian version of 15-item Patient Health Questionnaire (PHQ-15) [58, 59]. Participants were asked to rate how much they have been affected by the most common 15 somatic symptoms in primary care during the last 4 weeks (fatigue, gastrointestinal, musculoskeletal, pain, and cardiopulmonary symptoms). Each item is scored on a three-point Likert scale from 0 (“not bothered at all”) to 2 (“bothered a lot”). The total score can range between 0 and 30. Scores of 5, 10, 15, indicate cutoff points for low, medium, and high somatic symptom severity, respectively [58]. Within this sample, Cronbach’s α was 0.78 (T0) and 0.85 (T1).

*Alexithymia*. Alexithymia was measured using the Italian version of 20-item Toronto Alexithymia Scale (TAS-20) [60, 61]. Participants were asked to rate how much they agree or disagree with 20 items about feelings. Each item is scored on a five-point Likert scale from 1 (“strongly disagree”) to 5 (“strongly agree”). The total score can range between 20 and 100. A score of ≥ 61 is typically used as the cutoff for high alexithymic traits [62]. TAS-20 has three subscales for the affective (DIF, DDF) and the cognitive (EOT) factors. The DIF is composed of 7 items and measures the difficulty to discriminate between feelings and the bodily sensations of emotional arousal (e.g., “I am often confused about what emotion I am”). The DDF is composed of 5 items and measures the capacity to describe feelings to other people (e.g., “It is difficult for me to find the right words for my feelings”). The EOT is composed of 8 items and measures the tendency to focusing on concrete and factual details of external reality and to avoiding emotional nuances of emotional life (e.g., “I prefer talking to people about their daily activities rather than their feelings”). Within this sample, Cronbach’s α was 0.82 (T0) and 0.88 (T1).

### Statistical analysis

Preliminarily, we performed a power analysis to determine the sample size needed to detect a medium effect size. Power analysis was conducted using: (1) an estimated mean effect size of f = 0.25 and f^2^ = 0.15 for the ANCOVA repeated measures and hierarchical regression, respectively; (2) an alpha level of 0.05; and (3) a power of 0.95. According to statistical computing, a sample size of n = 66 and n = 172 was required for ANCOVA repeated measures and hierarchical regression, respectively. Power calculation was performed using the program G*Power 3.1 [63].

Data analysis was performed using SPSS 26.0 for Windows. Descriptive statistics were reported in terms of mean and standard deviation or frequencies. The level of significance was set at 95%. The reliability of the scales was measured using Cronbach’s α. A value of 0.70 and above is good, 0.80 and above is better, and 0.90 and above is best; values substantially lower indicate an unreliable scale [64].

A 2-step strategy was used for data analysis.

First, repeated-measures analysis of covariance (ANCOVA) was used to compare between-group differences in psychological variables that are based on repeated observations while controlling for a confounding variable. The repeated-measures ANCOVA included anxiety, depression, somatic symptoms, and alexithymia as dependent variables, the time points T0 and T1 as a within-subject factor, loneliness at baseline as a covariate, and loneliness related-groups at T1 as the between-subject factor. Because of the lack of cutoff scores for the UCLA Loneliness Scale, students were grouped based on the distribution of the scale scores at T1. The participants were divided into three groups: (1) low loneliness (*n* = 42, ≤ 25th; centile = 20–31; (2) middle loneliness (*n* = 95, 25th–75th; centile = 31.01–53.25): (3) high loneliness (*n* = 40, ≥ 75th; centile = 53.26–80). The Eta-squared (η^2^) was used as a measure of effect size. A standardized effect size of 0.01–0.05 is considered small, 0.06–0.14 moderate, and > 0.14 large [65].

Second, hierarchical regression was used to evaluate the relative and independent role of each factor in predicting loneliness after the COVID-19-related lockdown. The UCLA Loneliness Scale score at T1 was considered as the dependent variable and the independent variables were baseline (T0) and change (Δ) in PHQ-9 total score, GAD-7 total score, and TAS-20 subscale scores. Each variable at baseline and its change were entered as predictors in separate blocks to determine how well each variable predicted the outcome. Change in psychological variables (Δ) was expressed as the proportion of change from pre- (T0) to post- (T1) COVID-19-related lockdown based on baseline score and calculated as follows: {[(T1 – T0)/ T0] * 100}.

## Results

### Characteristics of the sample

No significant difference was found between recruited (*N* = 177, 95.67%) and excluded subjects (*n* = 8, 4.33%) for sociodemographic and COVID-19-related variables (data not shown). As expected, participants were mostly young females (*n* = 158, 89.3%; mean age = 22.53 years, *SD* = 2.18) and unmarried (*n* = 168, 94.91%). Only 7 (4%) students self-reported as being tested positive for COVID-19 in the previous months but no one had developed severe symptoms (see Table 1).

**Table 1:** Socio‐demographic and clinical characteristics of the study sample (N = 177)

Variable	Total sample N = 177
Age, mean (SD)	22.53 (2.18)
Gender, n (%)	
Men	19 (10.70)
Women	158 (89.30)
Marital status, n (%)	
Married	9 (5.09)
Unmarried	168 (94.91)
Tested positive for COVID-19, n (%)	
Yes	7 (4)
No	170 (96)

### Between‑group comparisons over time

The results of repeated measure ANCOVA are reported in Table 2.

**Table 2:** Comparisons of clinical characteristics before (T0) and after (T1) COVID-19 outbreak in loneliness-related groups

Variables, mean (SD)	Loneliness-related groups^a^			Time		Time*UCLA (T0)		Time*Group		Within-Groups comparisons
	A (Low) (*n* = 42)	B (Medium) (*n* = 95)	C (High) (*n* = 40)	*F*	η^2^	*F*	η^2^	*F*	η^2^	Post-hoc Bonferroni
*PHQ-9*										
T0	4.52 (3.19)	8.58 (5.29)	12.12 (6.74)	11.78**	0.07	10.16**	0.06	5.59**	0.06	A < C
T1	5.09 (3.66)	8.15 (5.06)	13.50 (6.44)							
*GAD-7*										
T0	4.88 (3.91)	7.44 (4.81)	9.20 (5.41)	22.16***	0.12	20.34***	0.11	9.29***	0.10	–
T1	5.24 (4.79)	7.04 (4.69)	10.82 (5.53)							
*PHQ-15*										
T0	6.33 (4.78)	8.73 (5.02)	10.52 (5)	0.005	–	0.16	0.001	0.88	0.01	–
T1	5.90 (4.51)	9.05 (5.20)	12.10 (5.97)							
*TAS-20*										
T0	35.19 (7.86)	43.48 (10.51)	47.95 (12.12)	8.93**	0.05	6.76*	0.04	9.92***	0.11	A < B, C
T1	32.83 (7.21)	44.99 (11.17)	53.17 (13.08)							
*DIF*										
T0	12.05 (4.19)	15.85 (6.61)	18.52 (6.18)	7.35**	0.04	6.22*	0.04	5.69**	0.07	A < B, C
T1	10.93 (3.56)	16.92 (6.41)	20.32 (7.42)							
*DDF*										
T0	10.14 (4.69)	13.29 (4.71)	14.97 (5.07)	2.18	0.01	3.19	0.02	5.28**	0.06	A < B, C
T1	8.90 (2.93)	12.73 (4.90)	15.87 (4.23)							
*EOT*										
T0	13 (2.69)	14.34 (3.57)	14.45 (4.52)	3.31	0.02	0.78	0.005	4.41*	0.05	A < B, C
T1	13 (3.19)	15.34 (3.52)	16.97 (4.67)							

*UCLA* University of California Los Angeles Loneliness Scale, *PHQ-9* Patient Health Questionnaire-9, *GAD-7* General Anxiety Disorder-7, *PHQ-15* Patient Health Questionnaire-15, *TAS-20* Toronto Alexithymia Scale-20, *DIF* Difficulties Identifying Feelings, *DDF* Difficulties Describing Feelings, *EOT* Externally Oriented Thinking

^a^Levels of loneliness at T1 as low (UCLA < 25th centile = 20–31), medium (UCLA 25th-75th centile = 31.01 – 53.25), and high (UCLA > 75th centile = 53.26—80)

All values are standardized regression weights. *p < .05; **p < .01; ***p < .001

The baseline level of perceived loneliness was used as covariate in order to investigate its influence on students’ psychological health during the COVID-19-related lockdown.

As expected, a significant effect of time on depression (*F* = 11.78, *p* = 0.001, η^2^ = 0.07), anxiety (*F* = 22.16, *p* < 0.001, η^2^ = 0.12), and alexithymia (*F* = 8.93; *p* = 0.003, η^2^ = 0.05), particularly the DIF component (*F* = 7.35; *p* = 0.007, η^2^ = 0.04), was found. The effects of time remained significant even after controlling for the covariate loneliness (T0) with effect size from small (η^2^ = 0.04) to moderate (η^2^ = 0.11).

Comparing groups with different levels of loneliness at T1, students with high loneliness (group C) reported higher depressive symptoms over time (*F* = 5.59, *p* = 0.004) than those with low loneliness (group A) (η^2^ = 0.06). Furthermore, students with either medium and high loneliness (groups B and C) reported a significant increase in the alexithymia total score (*F* = 9.92, *p* < 0.001, η^2^ = 0.11) as well as all its components DIF (*F* = 5.69, *p* = 0.004, η^2^ = 0.07), DDF (*F* = 5.28, *p* = 0.006, η^2^ = 0.06), and EOT (*F* = 4.41, *p* = 0.01, η^2^ = 0.05) compared to those with low loneliness (group A). In sum, students reported worsening depression, anxiety, and alexithymia over time. In students with high loneliness, these effects were more prominent.

### Predicting loneliness after COVID-19 outbreak

A hierarchical regression analysis has been performed to assess which variables contribute to explain the loneliness levels after the COVID-19 outbreak (UCLA Loneliness Scale at T1). The baseline (T0) and change (Δ) in depressive symptoms, anxiety symptoms, alexithymia dimensions served as independent variables (see Table 3).

**Table 3:** Hierarchical regression model examining baseline and changes in psychological variables as predictors of loneliness (T1)

Variables	Model 1			Model 2			Model 3		
	*B*	*SE*	β	*B*	*SE*	β	*B*	*SE*	β
PHQ-9 (T0)	1.36	0.17	0.58***	1.41	0.28	0.60***	0.82	0.27	0.35**
ΔPHQ-9	0.03	0.01	0.20**	0.03	0.01	0.17*	0.01	0.01	0.08
GAD-7 (T0)				− 0.06	0.34	− 0.02	− 0.11	0.33	− 0.04
ΔGAD-7				0.01	0.01	0.06	0.003	0.001	0.03
DIF (T0)							0.43	0.23	0.20
DDF (T0)							0.64	0.23	0.24**
EOT (T0)							0.25	0.28	0.07
ΔDIF							0.04	0.03	0.13
ΔDDF							0.09	0.03	0.21**
ΔEOT							0.07	0.03	0.15*
*R*^*2*^			0.29			.29			0.41
*F* for change in *R*^*2*^			32.79***			0.33			5.99***

*PHQ-9* Patient Health Questionnaire-9, *GAD-7* General Anxiety Disorder-7, *DIF* Difficulties Identifying Feelings, *DDF* Difficulties Describing Feelings, *EOT* Externally Oriented Thinking

All values are standardized regression weights. *p < .05; **p < .01; ***p < .001

In the first model, entering depressive symptoms (T0) and their change (ΔPHQ-9) significantly explained 29% of loneliness variance at T1 (β = 0.58, *p* < 0.001 and β = 0.20, *p* = 0.007, respectively). Adding anxiety (T0) and its change (Δ) (Model 2) did not contribute to explain a significant added variance (*R*^*2*^ change = 0.003). Adding alexithymia subscales in Model 3 produced an added 12% of explained variance, with DDF (T0), ΔDDF, and ΔEOT scores showing greater β values of 0.24, 0.21, and 0.15 respectively (*p* = 0.008, *p* = 0.01 and *p* = 0.05, respectively). Baseline depressive symptoms (PHQ-9 at T0) and some alexithymia components (particularly, DDF at T0, and higher ΔDDF and ΔEOT) significantly and independently predicted perceived loneliness after the COVID-19 outbreak and explained 41% of the variance in the final model.

## Discussion

Social distancing successfully slowed down the spread of the COVID-19 infection and relieved the burden on public health systems worldwide [66, 67]. At the same time, these population-wide measures affected psychological well-being and mental health as well as increased social isolation [68] and loneliness. In this longitudinal study, we aimed to evaluate the psychological correlates and predictors of perceived loneliness in college students during the COVID-19 lockdown. Our results are in line with previous reports from Italy, confirming a substantial proportion of mental health issues and high levels of loneliness among young adults during the COVID-19 pandemic [69, 70]. The main result was that students with higher depressive symptoms and alexithymic traits *before the lockdown* and whose levels of depression and alexithymia worsened *during the lockdown* were at higher risk of suffering from perceived loneliness. Our finding is consistent with a growing number of studies that have established links between loneliness and mental health [71]. For example, higher levels of loneliness were reported as strongly associated with greater depression and suicidal ideation [72, 73]. Mental health and loneliness are likely to be bidirectionally related as psychological distress may predict loneliness and, in turn, loneliness and social distancing the onset and the maintenance of psychological distress. The literature suggests that a significant part of the population was already lonely, socially isolated, or both before the COVID-19 pandemic [31, 74]. However, several studies suggest that loneliness increased by 20–30% during the pandemic, particularly among younger people, those with lower incomes, and those with chronic health conditions [75, 76]. Moreover, several COVID-19-specific stressors could further affect students’ psychological status, such as worries concerning one’s own and significant others’ health threatening issues, the financial stressors caused by the economic consequences of the pandemic, and the impact of a changing educational environment on the progress of their studies and future job market opportunities.

In our sample, suffering from depressive symptoms before COVID-19 and the aggravation of alexithymic aspects of difficulty talking about one’s feelings to others (change of the DDF component) as well as the adoption of a more concrete and factual thinking attitude (change of the EOT component) independently predicted perceived loneliness during the COVID-19 outbreak. A growing body of literature indicates alexithymia as a personality trait that greatly undermines relational quality [e.g., 43]. Evidence linking loneliness and alexithymia reveals that highly alexithymic individuals report poorer social support networks, fewer close relationships, and feeling of disconnection from others [44–46]. In addition, alexithymia is related to interpersonal indifference, which reflects low expectations for others, and a low desire to fulfill others’ expectations [77]. This line of research has led to studies examining the links between alexithymia, intimate communication, and relational health [44, 49, 78, 79]. Overall, this line of research points to intimate communication as one potential pathway by which alexithymia may contribute to relational problems and loneliness [80]. It should be noted, however, that the causal direction might be the inverse. The increase of alexithymia can be secondary to the COVID-related adverse events. Experiential avoidance, or the active avoidance of private experiences such as feelings, memories, and thoughts, could widely be associated with individual responses to cope with COVID-19 [41, 47, 48, 81]. Previous empirical research has shown that trauma exposure is strictly associated to the tendency to avoiding close contacts with affective experiences [e.g., 82–84].

In our sample, suffering from depressive before lockdown symptoms resulted to be a predisposing factor in perceived loneliness during the COVID-19 outbreak. The finding that loneliness is associated with depressive symptoms appears well established [6, 85–87]. Depressive symptoms may predict loneliness and, in turn, loneliness and social distancing the onset and the maintenance of psychological distress.

The present findings indicate that subjects with high levels of depression and alexithymia both at baseline and overtime are more prone to suffer from perceived loneliness. Therefore, one may speculate that alexithymic and depressive components may reinforce each other and contribute to feel more alone leading to perceive few benefits from social interactions and great confidence in social isolation. The capacity to express and communicate emotional and mental states to others is central to effective social interactions and interpersonal understanding such that it allows people to ensure adequate social relationships. Although other people are significantly present, individuals characterized by reduced abilities to understand and communicate emotions as well as low motivation, increased fears and negative feelings, low self-esteem, and sense of unworthiness, may feel disconnected from others and show poorly regulated distress responses. This may further hamper the social relationship through increased motivation to avoid others. Lifetime depression, alexithymia, and loneliness may be linked in a vicious circle. When this combination persists, it may affect individual vulnerability to chronic stress (allostatic load/overload) resulting in risk of developing physical and mental illness.

There is evidence that alexithymia can be successfully reduced with therapeutic interventions [88]. Most types of psychotherapy attempt to help people recollect, explore, and understand their emotions, and therefore consider working with emotions as a key mechanism for therapeutic transformation. Reaching students through early intervention is of paramount importance. Psychological interventions for mental health prevention and promotion may be a potentially effective means for several conditions. For example, counseling interventions could significantly reduce students' psychological distress by improving their ability to express and communicate emotional and mental states to others. Therefore, identifying students who are at risk for increased perceived loneliness is an important factor in targeting mental health promotion interventions for the most vulnerable students who would benefit from psychological treatment.

### Limitations and future directions

The longitudinal study design, the availability of pre-COVID-19 data, the high response rate, and the use of well-validated questionnaires are some major strengths of this study that has however some limitations. First, psychological symptoms and alexithymia were assessed only with self-report scales and not checked with multimethod assessment. Second, although expected in psychology students [89], the gender ratio was unbalanced with much more women than men. Third, several potential mediators were not controlled for, so that 60% of variance of perceived loneliness was explained by other factors (e.g., financial problems, forced co-dwelling, forced coming back home). Fourth, behavioral factors as the size of students’ social network, social support, and the use of social media were not controlled for whereas they are likely to influence perceived loneliness. Finally, the online assessment may bias the sampling procedure since personal motivations to participate can generate the overestimation of more distressed individuals or limit the participation of persons with less opportunity to use digital communication.

More longitudinal research with larger samples is required to verify if loneliness and psychological symptoms could be dissipated when the lockdowns ended or if the loneliness and psychological distress could continue despite communities reopening. There is also a need to develop research projects that can successfully distinguish between loneliness as a result of COVID-19 and loneliness caused by other factors.

## Conclusions

To conclude, mitigating the hazardous effects of COVID-19 on mental health is an international public health priority. It is of paramount importance to monitor how the COVID-19 pandemic and its long-term effects are affecting social interactions and psychological health of young students in order to provide appropriate psychological support. Indeed, social proximity and interpersonal interactions are relevant factors in developing and fostering social ties [90–92] and maintaining mental health [93]. Our longitudinal study suggests that college students who were depressed and had more alexithymic difficulty in expressing their feelings to others before the COVID-19 outbreak were those who experienced feelings of higher loneliness and isolation during the pandemic-related lockdown. Given the role played by perceived loneliness on health outcomes [1] and academic outcomes [94, 95], further longitudinal studies are needed to ascertain the medium to long term effects of loneliness, depression, and alexithymia. However, as the international guidelines point out [96, 97], planning for appropriate mental health services cannot wait for the long-term impacts of the pandemic to become apparent. Plans need to be formulated immediately to deal with the ongoing impacts of the pandemic. These plans should include youth-targeted actions within and outside higher education settings, including interventions for specific mental well-being and loneliness. The identification of subjects who are at risk of higher perceived loneliness and psychological distress is a chief factor for addressing mental health promotion to the most vulnerable individuals.

## Data Availability

The datasets used and/or analysed during the current study are available from the corresponding author on reasonable request.

## Abbreviations

ANCOVA: Analysis of covariance
DDF: Difficulty describing feelings
DIF: Difficulty identifying feelings
EOT: Externally oriented thinking
GAD-7: 7-Item generalized anxiety disorder scale
PHQ-15: 15-Item Patient Health Questionnaire
PHQ-9: 9-Item Patient Health Questionnaire
TAS-20: Toronto alexithymia scale
UCLA: University of California, Los Angeles

## Footnotes

1 Italian version of GAD-7 retrieved from www.phqscreeners.com/select-screener.
